# Functional Genomics Approaches to Elucidate Vulnerabilities of Intrinsic and Acquired Chemotherapy Resistance

**DOI:** 10.3390/cells10020260

**Published:** 2021-01-28

**Authors:** Ronay Cetin, Eva Quandt, Manuel Kaulich

**Affiliations:** 1 Institute of Biochemistry II, Goethe University Frankfurt-Medical Faculty, University Hospital, 60590 Frankfurt am Main, Germany; cetin@med.uni-frankfurt.de; 2 Faculty of Medicine and Health Sciences, Universitat Internacional de Catalunya, 08195 Barcelona, Spain; equandt@uic.es; 3 Frankfurt Cancer Institute, 60596 Frankfurt am Main, Germany; 4 Cardio-Pulmonary Institute, 60590 Frankfurt am Main, Germany

**Keywords:** chemotherapy resistance, cancer and drug vulnerabilities, functional genomics, RNAi and CRISPR screens

## Abstract

Drug resistance is a commonly unavoidable consequence of cancer treatment that results in therapy failure and disease relapse. Intrinsic (pre-existing) or acquired resistance mechanisms can be drug-specific or be applicable to multiple drugs, resulting in multidrug resistance. The presence of drug resistance is, however, tightly coupled to changes in cellular homeostasis, which can lead to resistance-coupled vulnerabilities. Unbiased gene perturbations through RNAi and CRISPR technologies are invaluable tools to establish genotype-to-phenotype relationships at the genome scale. Moreover, their application to cancer cell lines can uncover new vulnerabilities that are associated with resistance mechanisms. Here, we discuss targeted and unbiased RNAi and CRISPR efforts in the discovery of drug resistance mechanisms by focusing on first-in-line chemotherapy and their enforced vulnerabilities, and we present a view forward on which measures should be taken to accelerate their clinical translation.

## 1. Introduction

The administration of single anti-cancer drugs sooner or later selects for the occurrence and outgrowth of drug-resistant cancer cell populations, with therapy failure and disease relapse being the ultimate consequences. Focusing on chemotherapeutics, this led to the development of multidrug treatment protocols in which agents with different modes of actions are combined with the aim to suppress the occurrence of drug resistance. In hematological disorders, such as Hodgkin’s lymphoma and acute lymphoblastic leukemia, multidrug regimens, such as ABVD (doxorubicin-Adriamycin, bleomycin, vinblastine, and dacarbazine) or CHOP (cyclophosphamide, hydroxydaunorubicin, vincristine sulfate-oncovin and prednisone), when provided to patients with early-stage tumors, can result in 5-year progression-free survival of 80–98%, with many patients being cured. However, for most, if not all other solid and non-solid malignancies, therapy success with multidrug regimens remains to be the exception. 

**Figure 1:**
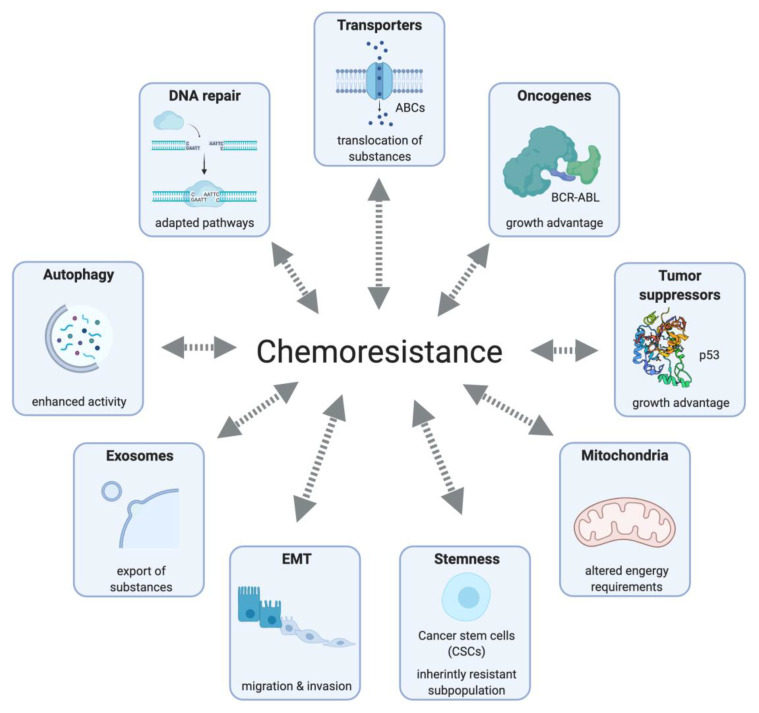
Mechanisms contributing to chemoresistance include molecule transporters that increase the drug efflux, reducing their intracellular concentrations; higher proliferation induced by oncogene activation or mutations in tumor suppressor genes; deregulation of apoptosis and metabolic reprogramming due to mitochondrial alteration; invasive phenotypes caused by overexpression of stem cell markers; existence of inherently resistant cell subpopulations which present a certain degree of quiescence and a high expression of stem cell markers, as well as drug efflux and anti-apoptotic proteins; elevated secretion of exosomes by tumor cells, which mediate the transfer of cargos that can promote resistance by several mechanisms (e.g., growth advantage, drug efflux); pro-survival function, mediated by increased autophagic activity; the activation of alternative DNA repair pathways.

Resistance can be restricted to a specific drug, or affect different drugs with independent modes of action, named multidrug resistance (MDR). However, even in non-solid tumors, chemotherapeutic multidrug regimes result in the appearance of drug-resistant cell populations, containing pre-existing (intrinsic) and newly acquired resistance mechanisms that can be mechanistically separated, as summarized in Figure 1.

Intrinsic resistance may be defined as the pre-existence of resistance mechanisms before therapy is initiated. The reasons are heterogeneous and include (1) the pre-existence of therapy-resistant cell populations; (2) low therapy tolerance of the patient or the occurrence of unbearable side-effects; (3) an inability of the therapy to reach its needed pharmacokinetic profile by means of altered absorption, distribution, metabolism, or excretion. In contrast to intrinsic mechanisms, acquired resistance may be defined by the appearance of drug-resistant cell populations containing secondary genetic modifications acquired during the course of therapy, ultimately, as with intrinsic resistance, leading to therapy failure. Acquired resistance mechanisms include, but are not limited to (1) increased rates of drug efflux or decreased rates of drug influx into the tumor cells, mediated by transmembrane transporters of drug uptake and/or efflux; (2) biotransformation and drug metabolism, mainly due to CYPs (Cytochromes P450s) in the tumor; (3) altered role of DNA repair and impairment of apoptosis; (4) role of epigenetics/epistasis by methylation, acetylation, and altered levels of microRNAs leading to alterations in upstream or downstream effectors; (5) mutation of drug targets in targeted therapy and alterations in the cell cycle and its checkpoints; (6) the tumor microenvironment. Importantly, cancers can become chemotherapy resistant by combinations of these mechanisms. For instance, the action of methotrexate depends on its active transport into cells through the reduced-folate transporter 1 (RFT-1), its conversion to a long-lived intra-cellular polyglutamate, and its binding to the dihydrofolate reductase (DHFR), which leads to the inhibition of the synthesis of thymidylate and purines and the induction of apoptosis. Cellular defects in any of these steps can lead to drug resistance. Mutations in RFT-1, amplification or mutation of DHFR, loss of polyglutamation, and defects in the apoptotic pathway have all been shown to lead to the loss of efficacy of methotrexate [1,2].

Anti-cancer drug resistance mechanisms, however, can be accompanied by the emergence of new and therapy-restricted vulnerabilities. For example, resistance can arise as a compensation for the effects of treatment due to the “addiction” of cancer cells to a specific oncogene. Functional genetic screens have been used to identify such acquired vulnerabilities in several cancer cell lines [3,4]. These dependencies, or collateral sensitivities, should be clinically exploited, as was demonstrated for drug-resistant melanoma. Through a “one-two-punch” strategy, treatment with vemurafenib (B-Raf inhibitor) leads to increased levels of reactive oxygen species (ROS) in resistant cells, rendering them particularly sensitive to the treatment with the histone deacetylase inhibitor vorinostat [5]. This exemplifies how comprehensive genetic mapping of drug resistance can be applied to synchronize a cancer cell population, enabling the collateral targeting of the resistance-associated vulnerability. In this review, we discuss targeted and unbiased gene perturbation efforts in elucidating the mechanisms behind chemoresistance and, whenever available, highlight associated vulnerabilities for a potential clinical exploration. 

## 2. Gene Perturbation Technologies

The term functional genomics comprises the levels of DNA (genomics and epigenomics), RNA (transcriptomics), proteins (proteomics), and metabolites (metabolomics). Each of them is of equal scientific relevance. This review will, however, exclusively discuss DNA (CRISPR) and RNA (RNA interference, RNAi) approaches in the discovery of resistance and vulnerabilities associated with chemotherapies. RNAi and CRISPR technologies enable unbiased high-throughput efforts that are invaluable tools to study gene function and establish phenotype-to-genotype relationships, complementing investigations of gene variants and knockouts in humans and mice by enabling the discovery of new vulnerabilities (Table S1). 

### 2.1. RNA Interference (RNAi)

**Figure 2:**
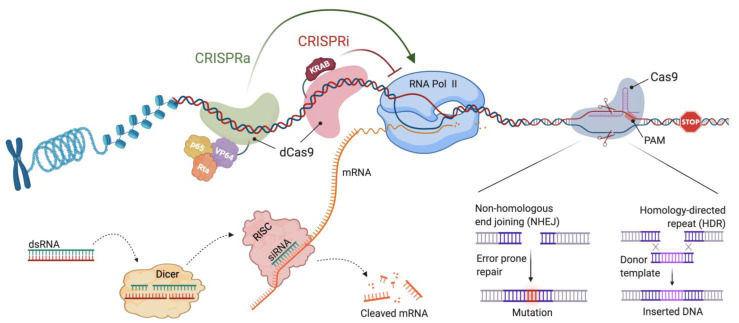
Mechanisms of RNAi and CRISPR. Gene knockdown can be induced by RNAi, triggered by endogenous miRNA, and exogenous siRNA or shRNAs. Pre-dsRNA molecules are trimmed into fragments of 20–25 nucleotides in the cytoplasm by Dicer. Their guide strand associates with the RISC complex that is then guided to target a bound RNA-complementary mRNA sequence, resulting in mRNA cleavage, degradation and stalled protein translation. To interfere with mRNA and protein abundance on the level of genomic DNA, CRISPR is used. Two partially-annealed RNA molecules, gRNA/tracrRNA, form a complex with Cas9, that scans DNA for PAM motifs with adjacent gRNA complementarity. Once bound, Cas9 will induce a DSB at the target site. DSBs are repaired by HDR or NHEJ repair pathways. Error-prone repair through NHEJ is favored, resulting in InDel-mutagenesis and the appearance of premature stop codons, collectively leading to gene knockout phenotypes. In combination with repair-templates, HDR is used for knock-ins. The CRISPR system with dCas9 can regulate genes by binding their promoters or regulatory elements. Transcriptional activation or inhibition through CRISPRa and CRISPRi is achieved by fusing transcriptional activator or repressor domains to dCas9.

In 1998, it was shown that the injection of double-stranded RNA (dsRNA) molecules into Caenorhabditis elegans could potentially silence any gene [6]. The discovery of the RNAi mechanism made it possible and feasible to study gene functions at scale. Soon after its development, the RNAi technology was established for mammalian cells to downregulate specific gene products [7]. RNAi is a process of silencing gene translation by cleavage or suppression of messenger RNA (mRNA) through the use of mRNA-complementary short RNA sequences (Figure 2). RNAi can be triggered by endogenous microRNAs (miRNA), small interfering RNAs (siRNA), or synthetic exogenous short hairpin RNAs (shRNA) [8,9]. Primary miRNA and shRNA (pre-miRNA or -shRNA) are expressed and processed by the Drosha protein in the nucleus before they are exported into the cytoplasm [10,11,12]. In the cytoplasm, pre-miRNA, pre-shRNA, or exogenous dsRNA are trimmed into double-stranded RNA molecules of appropriate sizes (19–21 nucleotides) by an endonuclease enzyme called Dicer, into miRNA, shRNA, and siRNA, respectively [13]. Once cleaved, they associate with the RNA-induced silencing complex (RISC), after which the argonaute protein (part of the RISC complex) cleaves the passenger strand of the dsRNA molecule, resulting in an active RISC complex, loaded with a single-stranded RNA (ssRNA) molecule [14]. The ssRNA is then used as a sequence-dependent guide to target complementary mRNA sequences, resulting in mRNA degradation and stalled protein translation (Figure 2). The combination of several siRNAs or shRNA allows the simultaneous targeting of a large number of genes in an unbiased format [15]. RNAi screens have been widely used, not only in cancer cells, to elucidate the proliferation and survival functions of genes in specific cell lineages, as well as to connect gene function to specific drug resistance mechanisms [16,17,18].

### 2.2. Clustered Regularly Interspaced Short Palindromic Repeats (CRISPR)

CRISPR and their associated proteins (Cas) are an adaptive immune system evolved in Bacteria and Archaea [19], by which sequence-information of viruses (bacteriophages) is genetically encoded into the host cell and used for pathogen defense. After characterizing the molecular mechanisms involved in sequence integration and pathogen defense, it was apparent that by changing the directionality of the CRISPR/Cas system to recognize DNA from other species, a genome-editing tool with unprecedented target range would be created [20]. Since then, multiple CRISPR/Cas systems have been described [21,22], their Cas nucleases characterized, engineered and applied to edit the genomes of several organisms, including bacteria, fungi, plants, worms, and humans [23,24,25,26,27,28,29,30,31]. At its core, genome editing with CRISPR/Cas requires three essential components: a guide RNA (gRNA), a tracer RNA (tracrRNA), and a Cas protein with nuclease activity. Cas9 from Streptococcus pyogenes (SpCas9) was the first to be identified and is the most widely used nuclease [29,32]. Remarkably, even though multiple other Cas-enzymes have been characterized in great detail, the first discovered SpCas9 remains to be the most robustly performing Cas-nuclease. When co-expressed, gRNA/tracrRNA and Cas will form a ribonucleoprotein (RNP) complex that scans the target DNA for Cas-binding sites, so-called protospacer adjacent motifs (PAM). These short-sequence motifs are highly Cas-dependent (with NGG being the PAM of SpCas9), and their overall number per genome represents the Cas-specific target range. Once bound to the PAM sequence, a conformational change in Cas enables Watson–Crick base pairing between gRNA and target DNA. In the case of perfect annealing, the two catalytic centers of SpCas9 will induce a double-strand break (DSB) at the target site (Figure 2), and, depending on the used Cas enzyme, leave blunt (SpCas9) or sticky ends (Cas12a) behind [20]. Although perfectly matched target sequences (on-targets) are favored, cutting at sequences with mismatches (off-targets) is possible and is thus a major concern [33], especially for therapeutic applications where off-target cutting must be avoided. Two major repair pathways have been described for Cas-induced DSB, homology-directed repair (HDR) and non-homologous end joining (NHEJ) [34]. Since both alleles are disrupted simultaneously, HDR is incapable of repairing the lesions due to the lack of a repair template, leading to the error-prone repair through NHEJ (Figure 2). In line with this, the global profiling of repair outcomes demonstrated that insertions and deletions (InDels) are the most frequent events [35], with larger deletions and complex repair events occurring less frequently [36,37]. However, single gRNA-mediated gene targeting results in an out-of-frame probability of 66.6% for any given gene. For most genes, this will result in a premature stop codon and a quantifiable knockout (KO) phenotype [38]. Importantly, it should be noted that it is currently unclear if the presence of truncated protein fragments represents a confounding factor for the genotype-to-phenotype correlation. In contrast to the occurrence of NHEJ-dependent InDels, HDR is the preferred pathway for targeted gene knock-ins (KI) [39,40] (Figure 2). Since approaches to perform highly efficient KI have been reviewed before, we refer the interested reader to these references [39,40]. The ease of directing the CRISPR/Cas system to almost any region in a given genome makes it a well-suited system for high-throughput functional genomic studies for the unbiased correlation of genotypes and phenotypes [41].

In contrast to wild-type Cas nucleases, partially or fully inactive Cas-enzymes have been generated and linked to effector domain-containing proteins [34]. The CRISPR system with nuclease-deficient Cas9 (dead Cas9, dCas9), incapable of DNA cleavage, retains its DNA binding ability and, thus, can regulate genes by binding their promoters or regulatory elements that close to their transcription start site (TSS). Transcriptional regulation is achieved by fusing a transcriptional activator or repressor domain to dCas9 [42,43,44,45] (Figure 2). CRISPR interference (CRISPRi) comprises an inactive dCas9-repressor complex with a gRNA designed to target the 3′ region of the TSS. Transcriptional repressor complexes, such as KRAB (Kruppel-associated Box) domains, result in specific and the tunable down-regulation of gene expression. On the other hand, CRISPR activation (CRISPRa) enhances gene expression by a dCas9 fused to transcriptional activators as, for example, VPR (VP64, p65, Rta fusion) domains [46]. CRISPRa allows for enhancing gene transcription at endogenous gene loci by targeting activation complex 5′ to a gene’s TSS. Both concepts, regulating gene expression through the recruitment of transcriptional activators or inhibitors, are suited for genome-wide screens and are of particular importance when DSB and InDel mutagenesis are unlikely to result in desired phenotypic consequences, e.g., non-coding RNAs or intergenic regions [44].

### 2.3. Comparison of RNAi and CRISPR Gene Perturbation Technologies

siRNAs can be generated in a cell from a vector encoding shRNAs and processed via the endogenous small RNA biogenesis pathway [9]. The use of several distinct siRNAs or shRNA sequences, in the forms of libraries, allows for the simultaneous and unbiased targeting of a large number of genes. Similarly, CRISPR screens can be performed by using gRNA libraries. These unbiased approaches are powerful tools to study genotype-to-phenotype relationships in, for example, elucidating drug resistance mechanisms. The RNAi-processing components act mainly in the cytoplasm with associated gene knockdowns, being independent from accessibility to and the number of chromosomes, making RNAi screens better suited for investigating polyploid cell models, such as cancer cells, with Cas9 binding and activity being negatively affected by chromatin structure and the ploidy status of a cell [47]. However, computational approaches have been established to correct CRISPR-derived data for the ploidy status of cell. RNAi screens do not require the expression of additional exogenous proteins, such as Cas9 or effector proteins, though they also require long-term selection to generate clonal cell lines. It is worth mentioning that RNAi is highly susceptible to off-target effects, leading to the knockdown of undesired genes and representing a major confounding factor in unbiased RNAi screens [48,49]. In line with this, direct experimental comparisons of RNAi or CRISPR screens identified the CRISPR system to outcompete RNAi with higher on-target and fewer off-target effects [48,50]. The CRISPR method generates complete gene knockouts through the InDel mutations, whereas RNAi mostly results in the partial suppression of the targeted gene transcript [51,52]. Therefore, the identification of phenotype-relevant genes becomes less error-prone when they are targeted with CRISPR compared with RNAi. Double-stranded siRNAs are also recognized as foreign molecules by a cell’s innate immune system and can stimulate immune responses. This potentially confounds the results obtained by si/shRNA investigations and can lead to phenotypic characterizations that are indirect or unrelated to the targeted gene [53,54], exemplified by the fact that CRISPR/Cas9 libraries were able to identify more essential genes in a direct experimental comparison of CRISPR/Cas9 and RNAi screens for essential genes [55]. Despite increased performance, targeting a desired sequence is more limited with the CRISPR method, as only sequences adjacent to PAMs can be targeted by CRISPR, whereas RNAi in principle can target any mRNA sequence. This limitation, however, can be overcome by using modified Cas enzymes, which have improved targeting ranges [34,56,57]. Lastly, Cas9-induced DNA damage might result in false-positive or false-negative results, and potentially cellular toxicity. An apparent phenomenon when targeting amplified genomic regions or performing CRISPR experiments in TP53-positive cells [58,59].

Compared to other methods that enable gene regulation, CRISPRi and CRISPRa have the advantage of design simplicity, high specificity, and few off-target effects, concertedly overcoming the limitations of RNAi. Particularly when functional investigations of non-protein-coding RNAs, such as long noncoding RNAs (lncRNA), miRNA or gene regulatory elements is desired, the CRISPRi/a system outcompete RNAi and CRISPR knockout approaches [60,61,62,63]. CRISPRi/a shows high specificity with minimal off-target effects, thus minimizing unspecific phenotypes and InDel heterogeneity, explained through the DNA-gRNA mismatch-sensitive activity of CRISPRi/a and the targeting of non-coding regions surrounding the TSS [64]. The knockdown efficiency of CRISPRi is higher than RNAi, but variations in gRNA efficiency and clonal variations are still possible and also observed [64]. The inhibition or activation of gene transcription can also be achieved by changing the epigenetic profile of DNA surrounding a gene’s TSS [65]. Chromatin structure and its “compactness” can negatively affect dCas9 binding, as previously observed for Cas9, a phenomenon that RNAi circumvents [47]. Furthermore, the annotation of a TSS is another possible confounding factor that determines the efficiency of CRISPRi/a. RNAi can be designed to specific splice variants or RNAs with mutations, which is not yet generally possible with CRISPRi/a, for which the targeted depletion of single transcripts still represents a major bottleneck that needs to be overcome [66]. Moreover, CRISPRi/a is poorly efficient for genes that are controlled by more than one TSS, or TSS’ that control more than one gene, as in the case of bi-directional promoters [67]. Overall strengths and weaknesses of RNAi and CRISPR technologies are compared in Table 1.

## 3. Mode of Action, Resistance Mechanisms and Vulnerabilities of Chemotherapeutics

### 3.1. Alkylating Agents/Platinum-Based Agents

Alkylating agents are anti-cancer drugs that add an alkyl group to the guanine base of DNA, resulting in DNA crosslinking and the inhibition of transcription. Alkylating agents are classified as monofunctional or bifunctional agents that cross-link one or two strands of the DNA, respectively. Alkylating agents cause cytotoxic, mutagenic, and carcinogenic effects by preventing DNA replication and RNA transcription. This action occurs in all cell types although rapidly dividing cells, such as cancer cells, being affected the most. However, some cell types, such as reproductive, endothelial or hematopoietic cells, also divide rapidly and toxicity in these cell types accounts for the common side effects of alkylating agents. Alkylating agents are classified as (1) nitrogen mustards (mechlorethamine, cyclophosphamide, ifosfamide, melphalan, chlorambucil, bendamustine); (2) alkyl sulfonates (busulfan, hepsulfam); (3) aziridines/ethyleneimines (thiotepa, mitomycin C, triethylenemelamine); (4) Hexitol epoxides (di-epoxide 1,2:5,6-dianhydrogalactitol (DAG) and 1,2-dibromodulcitol (DBD)); (5) nitrosoureas (carmustine, lomustine); (6) triazenes and hydrazines (dacarbazine, temozolomide, procarbazine); (7) the platinum-containing antineoplastic agents (cisplatin, oxaliplatin, carboplatin) [81]. Platinum-based antineoplastic agents are often associated with alkylating agents, but they have distinct characteristics. They do not alkylate DNA, but their chemotherapeutic mechanism appears to be similar to that of conventional DNA-modifying alkylating agents.

Similar to alkylating agents, DNA adducts of platinum-based agents inhibit DNA replication, thereby causing the inhibition of mRNA and protein synthesis, triggering cell death by the induction of apoptosis [82,83]. Cisplatin (cis-diamminedichloroplatinum(II)/CDDP) is a widely used platinum-based anti-cancer agent. It is a neutral, square planar inorganic compound of platinum (II) that is coordinated with two labile chloride and two stable ammonia groups in the cis-configuration [84]. Cisplatin enters the nucleus in its neutral form and undergoes aquation, rendering it highly reactive to DNA. Cisplatin primarily modifies the N7 position of the guanine, forming DNA-cisplatin adducts, which are critical for cisplatin-induced cytotoxicity [85,86]. As a result, cisplatin inhibits DNA replication and transcription and induces DNA DSBs that collectively cause cell death. Cisplatin is clinically approved as an anti-tumor drug for the treatment of several cancers, including breast cancer, pancreatic cancer, bladder cancer, and non-small cell lung cancer (NSCLC). After an initial good response to cisplatin treatment, tumors can quickly adapt and become resistant, resulting in therapy failure and disease relapse. A number of cisplatin resistance mechanisms have been described, including (1) decreased uptake/increased release of the compound; (2) inactivation of the drug; (3) enhanced DNA repair; (4) decreased apoptosis; (5) upregulated autophagy (reviewed in [87]).

A well-known characteristic of platinum-based drug-resistant cells is enhanced DNA repair [88,89]. The breast cancer gene (BRCA) pathway is involved in DNA repair and DNA damage response with BRCA-deficient cancer cells being more sensitive to platinum-based agents due to their reduced ability to perform DNA repair by homologous recombination (HR) [90,91]. As discussed above, genome-scale gene perturbation screens allow for the unbiased determination of genes, pathways or networks involved in cellular resistance. To do so, Batz and colleagues performed a genome-scale siRNA screen, in which 20,000 human genes were targeted in cervical, lung, and ovarian cancer cell lines, with the aim to identify genes that enhance the sensitivity of the cells to cisplatin [92]. The screen was performed in TP53-positive and -deficient cells and they identified the lack of BRCA1/2 and RAD6/RAD18-dependent DNA repair pathways to enhance the sensitivity of TP53-deficient tumor cells to cisplatin. The hits from this screen mainly included genes that play key roles in DNA repair and cell-cycle checkpoints. While the DNA repair gene REV1 and the checkpoint genes ATR and CHEK1 were found to be cisplatin-sensitivity enhancers, the most prominent hit genes from the screens were members of the BRCA pathway (BRCA1, BRCA2, BARD1, RAD51, and SEM1) with their silencing, resulting in the increased cisplatin-dependent cytotoxicity of TP53-deficient cells. This study agrees well with multiple independent studies that also identified BRCA-deficient cancer cells to be more sensitive to DNA damaging agents, such as cisplatin [90,91,93,94]. However, cisplatin resistance still occurs in BRCA-mutated cancers that are independent of BRCA-mediated HR restoration. In order to understand this phenomenon, a loss-of-function genome-wide screen in the BRCA2-mutant ovarian cancer cell line PEO1 was performed [95]. In this screen, 28,000 genes were targeted and the loss of the nucleosome remodeling factor and SNF2/RAD54 helicase family protein CHD4 was found to trigger cisplatin resistance. Interestingly, the study proposes the loss of CHD4 expression to prime cisplatin resistance through an RAD18-dependent mechanism, rather than an HR-dependent mechanism, with its molecular mechanism still being elusive.

In an earlier study, the deficiency of the DNA mismatch repair pathway has been shown to contribute to cisplatin resistance [96]. A whole-genome CRISPR screen to investigate cisplatin resistance mechanisms in bladder cancer identified MSH2 as the most enriched gene and the mismatch repair pathway as the most significantly enriched pathway [97]. Gene ontology (GO) enrichment analysis on the 48 most-enriched genes of the screen identified the mismatch repair pathway as a key pathway to mediate cisplatin resistance. Moreover, down-regulation of the mismatch repair pathway components MSH2 and MLH1, the top hit genes from the screen, increased resistance to cisplatin in cell culture. Additionally, shRNA-mediated knockdown of MSH2 in MGHU4 and 253J bladder cancer cell lines increases resistance to cisplatin, validating their approach and hit genes.

Cancer cells often depend on the G2/M cell cycle checkpoint to arrest in response to genotoxic stresses, thereby escaping the induction of apoptosis and cell death. Thus, cancer chemotherapy relies on the G2/M checkpoint mechanism [98]. CRISPR knockout screening in malignant pleural mesothelioma (MPM) cells with a focused gRNA library, targeting 763 human kinases, identified G2/M checkpoint kinases, including WEE1, whose deficiency sensitizes MPM cells to cisplatin/pemetrexed (MTA) combinatorial therapy [99]. The authors demonstrated that knockout or chemical inhibition of the WEE1 kinase re-sensitizes MESO-1 cells, an MPM cell line, to cisplatin/MTA treatment. Mechanistically, the inhibition of WEE1 reverses chemotherapy-induced G2/M cell cycle arrest by preventing the inactivation of CDK1 which, in its active form, causes premature mitotic entry, enhanced DNA damage and apoptosis.

The mTOR pathway is well described as a critical circuit of anti-cancer agent sensitivity [100,101]. Geoerger et al. demonstrated that rapamycin has an additive cytotoxic effect with cisplatin in medulloblastoma [102]. In line with this, Guerreiro and colleagues conducted an arrayed kinome-wide survival RNAi screen in the presence and absence of low dose cisplatin in order to identify genes involved in cisplatin resistance in medulloblastoma [103]. In this study, DAOY cells were screened with an arrayed library which targets 719 human kinase genes with 2157 specific siRNAs. Twenty-four top hits from the screen were identified, as their inhibition increased the sensitivity of the cells to cisplatin treatment. Six of those genes were reported as contributing to both cisplatin resistance and cell proliferation in medulloblastoma cells. Their validation experiments indicate that the silencing of ATR, LYK5, MPP2, PIK3CG, PIK4CA, and WNK4 increases the effects of cisplatin in medulloblastoma cell lines. Focusing on two of these genes, namely the PI3K isoform p110g and LYK5, the group was able to show that these genes have important roles in medulloblastoma chemoresistance and proliferation by a mechanism involving mTOR activation.

The genome-wide CRISPR loss-of-function screen also identified genes taking part in cisplatin resistance in melanoma [104]. In this study, the A375 melanoma cell line was combined with the very first CRISPR library (GeCKO.v2) that targets 99.4% of all human genes. Subsequent to the screen, the group performed a GO enrichment analysis and retrieved several processes responsible for cisplatin sensitivity (protein translation, mRNA catabolic processes, SPR–dependent cotranslational protein targeting to membrane, mitochondrial respiratory chain complex assembly) and cisplatin resistance (negative regulations of cellular catabolic process, proteasome-mediated ubiquitin-dependent protein catabolic process, regulation of cellular protein localization). Interestingly, neither influx or efflux, nor DNA repair pathways were found. Instead, ZNRF3, RNF7, and UBE2F were identified as genes whose disruption decreases cell survival upon cisplatin treatment. ZNRF3 is an E3 ubiquitin ligase and a negative regulator of the Wnt/β-catenin signaling pathway, while NF2 is a known tumor suppressor found to sensitize A375 melanoma cells to cisplatin upon its disruption [105]. If NF2 sensitization depends on its function in PI3K, mTOR, Ras, LIN28B, and EGFR pathways, or is due to the decreased cell cycle time of NF2-negative cells, remains to be elucidated [106].

After the formation of the DNA-cisplatin adducts, the main downstream event is triggering apoptosis. Cisplatin resistant cancer cells usually display impaired apoptotic cell death machinery. Enhancing or re-establishing apoptosis in resistant cells is, thus, a promising approach to cancer treatment. Focused siRNA screens targeting genes associated with ubiquitin and ubiquitin-like signaling have been performed by MacKay et al. to address the relationship between these genes and cisplatin [107]. They used a siRNA library, targeting 1067 genes, which are validated and computationally predicted to be associated with ubiquitin or ubiquitin-like signaling machinery. U2OS osteosarcoma cells were used to conduct the screen, plus a panel of cancer cell lines, such as HCT116 (colon), HeLa (cervix), PC3 (prostate), ZR.75.1 (breast), MDA-MB-231 (breast), and PEA1 (ovary), which were used to validate the hits from the screen. HOIP (RNF31) has been identified as a novel anti-apoptotic regulator of cisplatin cytotoxicity and it is required for resistance to cisplatin. Cells with depleted HOIP have been shown to have increased cisplatin cytotoxicity. They demonstrated that enhanced Caspase-8-mediated apoptosis is responsible for this cisplatin sensitization and it is mainly dependent on ATM-mediated DNA damage checkpoint activation. Collectively, the HOIP-mediated anti-apoptotic pathway has been demonstrated in this study. Besides impaired apoptosis, the precise functioning of other cell death mechanisms is important in cancer. Resistance to anoikis, an anchorage-dependent cell death, is a common feature of and causes chemoresistance in cancer cells [108]. Yamanoi and colleagues conducted a functional genomics screen using an shRNA library to determine drivers of anoikis resistance in ovarian cancer cells [109]. The OVCA420 ovarian cancer cell line was transduced with a library of 80,000 shRNAs targeting 15,000 human genes. The suppression of ABHD2, ELAC, and CYB5R3 was found to increase resistance to anoikis. In addition, the downregulation of ABHD2 enhances not only anoikis resistance but also cisplatin resistance in ovarian cancer through the phosphorylation of ERK and p38. Overall, hits from functional genetics screens studying cisplatin resistance show variations, but this can be improved by performing well-established gene perturbation screens in large panels of cell lines.

### 3.2. Topoisomerase Inhibitors

Topoisomerases act on DNA to resolve structural and topological issues during DNA replication and transcription through a DNA strand braking reaction that forms a covalent phosphotyrosine link and simultaneously breaks a DNA phosphodiester bond. Two types of DNA topoisomerases exist: type I enzymes break the DNA strands one at the time, while type II enzymes break both strands of the DNA double helix in parallel. Type I and II enzymes are further subdivided into IA, IB, IIA, and IIB based on structural and mechanistic similarities. By stabilizing the covalent DNA-topoisomerase intermediate, an enzyme required for cellular homeostasis is hijacked to induce DNA-damage, resulting in replicative and transcriptional stress, cytotoxicity, and cell death. Based on their mode of action, topoisomerase inhibitors are grouped into 6 categories: (1) competitive substrate inhibition—inhibition of the topoisomerase active site to prevent DNA binding [110]; (2) formation of “topoisomerase poisons”—formation of a locked ternary DNA-topoisomerase-inhibitor complex to prevent DNA re-ligation [111]; (3) competitive inhibition of the ATP binding pocket—interference of ATP-hydrolysis of type II enzymes; (4) “unbindable” DNA due to inhibitor binding to target DNA [112,113]; (5) inhibitor binding to the DNA-topoisomerase complex to prevent DNA cleavage [113,114]; (6) inhibition of ATP hydrolysis—after DNA strand passage occurred, type II enzymes are prevented from releasing the DNA [115,116,117]. Clinically relevant topoisomerase inhibitors mostly fall into category 2, “topoisomerase poisons”, and are either camptothecin derivatives (topotecan, irinotecan and belotecan), anthracycline derivatives (doxorubicin, epirubicin, valrubicin, daunorubicin and idarubicin), or epipodophyllotoxin-derived agents, such as etoposide and teniposide. Camptothecin derivatives act on type I topoisomerases, causing cytotoxicity by inducing DNA DSBs and preventing DNA replication [118]. In contrast, anthracycline derivatives or epipodophyllotoxin-derived agents act on type II topoisomerases by intercalating into the topoisomerase-bound and cleaved DNA and by direct binding to topoisomerase II, respectively, causing a stable ternary complex that is incapable of DNA re-ligation [119,120]. The first topoisomerase inhibitors that were used for cancer therapy are the anthracycline derivatives doxorubicin, epirubicin, valrubicin, daunorubicin, or idarubicin. Anthracycline derivatives have indications that include the treatment of breast cancer, leukemias, sarcomas, carcinomas, lymphomas, as well as urinary bladder cancer. Their topoisomerase “poisons” mode of action and the blocked re-ligation of the cleaved DNA strands is likely to be the mechanism underlying cell death induced by anthracycline derivatives. Moreover, the intracellular enzymatic reduction of the anthracycline quinone ring and the formation of iron anthracycline complexes generates free radicals that cause membrane lipid peroxidation and intrinsic and extrinsic apoptosis pathway activation [121]. Cardiomyocytes, compared to other tissues, have a lower activity against antioxidants, consequently leading to a selective cardiac toxicity profile of anthracycline derivatives [122]. In contrast, the camptothecin derivative topotecan is approved for small cell lung cancer (SCLC) and, together with the alkylating agent cisplatin, for the treatment of cervical cancer, whereas Irinotecan is approved for metastatic colon or rectal cancer, plus the semi-synthetic camptothecin derivative Belotecan being approved for SCLC and ovarian cancer.

Several resistance mechanisms are described for topoisomerase inhibitors, that can broadly be defined by removing the drug or the drug target, and/or by a changed cellular response to the drug or the interference with DNA damage sensing. Mechanistically, topoisomerase resistance includes (1) an altered topoisomerase expression profile; (2) topoisomerase mutations that reduce inhibitor affinity; (3) MDR protein expressions and their activity; (4) decreased formation of the cytotoxic metabolite SN38 through reduced expression or activity of carboxylesterase; (5) the increased expression of sulfhydryl proteins such as glutathione and the glutathione-dependent protein (collectively also reviewed in [123,124,125]). MDR is of particular importance as it generates cross-resistance to unrelated cancer drugs by intrinsic and acquired mechanisms, caused by increased expression of MDR proteins 1, 2 and 3 (MDR1/2/3) [126]. MDR1, also known as permeability glycoprotein 1 (P-gp) or ATP-binding cassette sub-family B member 1 (ABCB1) or cluster of differentiation 243 (CD243), functions as an ATP-consumption pump to stimulate the efflux of cytotoxic compounds [127,128]. MDR1 overexpression is therapeutically actionable with competitive and non-competitive blockers of MDR1 function, both resulting in reduced efflux and in increased intracellular concentrations of the drug, collectively leading to increased chemotherapeutic-induced toxicity of cancer cells [129]. However, competitive and non-competitive blockers of MDR1 function are not exempt from occurring resistance, questing this as a viable approach to overcome topoisomerase inhibitor resistance.

Genome-wide screens contributed to the understanding of topoisomerase inhibitor resistance mechanisms linked to drug or drug target removal. As such, individual drugs and dual drug combinations of the R-CHOP (R-Rituximab, C-Cyclophosphamide, H-Doxorubicin Hydrochloride (Hydroxydaunomycin), O-Vincristine Sulfate (Oncovin), P-Prednisone) regimen were applied to quantify cross-resistance in diffuse large B-cell lymphoma cells by CRISPRa and CRISPRi screens. In line with previously described resistance mechanisms, the ABC multidrug resistance transporter genes ABCC1, ABCG2, and ABCB1 were identified in the CRISPRa screen, whereas the Hydroxydaunomycin-target gene itself, TOP2, was identified in the CRISPRi screen to induce Hydroxydaunomycin resistance [130], which is consistent with data derived from a previous CRISPR knockout study [38]. These findings are also supported by a pool-based RNAi screen for doxorubicin resistance-causing factors that identified TOP2A to be the major determinant of drug response [131]. Of note, the Sorger group provided experimental evidence that R-CHOP drugs exhibit low cross-resistance [130], supporting the hypothesis that anti-cancer therapies can be rationally designed on the ground of non-overlapping drug resistance mechanisms. Building on these studies, the Lamba group used the CRISPR knockout library “Brunello” in chronic myelogenous leukemia (CML) K562 cells combined with etoposide exposure and also identified the genetic ablation of ABCC1 to increase etoposide-dependent toxicity [132]. Most importantly, the same group identified the DNA damage repair helicase RAD54L2 and showed that the expression level of ABCC1 and RAD54L2 is associated with etoposide resistance, event-free survival (EFS) and overall survival (OS) in childhood acute myeloid leukemia [132,133], providing evidence that in vitro CRISPR screening hits have direct clinical relevance.

Despite drug and drug target removal, changes in a cell’s response to the drug itself, including the interference of DNA damage sensing are also implicated in resistance to topoisomerase inhibitors. In line with this, Wijdeven et al. performed an insertional mutagenesis screen in haploid HAP1 cells using a gene trap approach to search for doxorubicin resistance-causing genes and identified the ABC transporter ABCB1, the Cul3-dependent ubiquitin ligase adaptor KEAP1, as well as the multi-subunit chromatin-remodeling SWI/SNF complex [134]. This screening approach identified doxorubicin resistance mechanisms linked to reduced DNA DSB formation and enhanced DNA repair. An independent study conducted by Borys et al. generated a CRISPR library targeting 330 putative p53-target genes and identified the cell cycle inhibitor CDKN1A and the solute carrier family 30 member 1SLC30A1 to promote growth [135]. Subsequently, the same group used the CRISPRi (KRAB domain) approach to transcriptionally downregulate genes associated with thousands of p53 binding sites and asked which are relevant for promoting growth during doxorubicin exposure. CDKN1A target sites were among the most enriched, with the vast majority of sites located in a 15kb window surrounding the CDKN1A TSS [135]. However, at least one significantly enriched site was more than 250kb away from the CDKN1A TSS, suggesting a role of non-protein-coding sequence elements in doxorubicin resistance. In line with this, Wegner et al. generated the largest to-date CRISPR library targeting coding and non-coding regions based on Cas9′s gRNA-sequence preferences and applied the reagent to doxorubicin-exposed hTERT-immortalized RPE1 cells [41]. The Leukotriene C4 G-protein-coupled eicosanoid receptor CysLTR2, that was previously reported to induce doxorubicin resistance by preventing the accumulation of reactive oxygen species [136], was the strongest protein-coding hit gene. However, 50.3% of retrieved gRNAs accounted for non-protein-coding regions with eight gRNAs targeting positions of the a-kinase anchor protein AKAP6 and the aspartoacylase ASPA2 genes, of which AKAP6 was previously linked to doxorubicin sensitivity [137]. Moreover, Wegner et al. identified predicted promoter and enhancer sequences, lincRNAs, pseudogenes, as well as CTCF binding sites, further supporting a hitherto underappreciated role of non-coding sequences in doxorubicin resistance [41].

Besides identifying genes that contribute to drug resistance, unbiased gene perturbation screens are also powerful approaches to identify genes on which a drug-resistant cancer cell depends on. These approaches, however, require large panels of drug-resistant cell models that are sparse, and the conduction of genome-scale screens to comprehensively map these dependencies. Bypassing these limitations, Ha et al. performed a proof-of-concept study and CRISPR-targeted the MDR1 gene in doxorubicin-resistant breast cancer cells and demonstrated that this edit restored doxorubicin susceptibility [138]. Moreover, Wang et al. studied the cell membrane-tethered urokinase plasminogen activator receptor (uPAR), a gene frequently overexpressed in cancer cells, and demonstrated that its genetic ablation in endocervical and colorectal adenocarcinoma cell lines reduced resistance to 5-FU, cisplatin, docetaxel, and doxorubicin [139]. CD44, yet another cell surface-located glycoprotein, was genetically removed from multidrug resistant osteosarcoma cells by CRISPR, resulting in inhibited migration and invasion activities, but also to enhanced doxorubicin sensitivity [140], collectively demonstrating the power of unbiased and targeted CRISPR approaches and suggesting a central role of cell surface-located proteins in drug-resistant collateral sensitivity.

### 3.3. Mitotic Inhibitors

Cells exposed to inhibitors of mitosis are generally characterized by a permanent cell cycle arrest that is mechanistically linked to the inability of actin or tubulin (or its polymeric form, microtubules) to polymerize or depolymerize [141], resulting in chromosome misalignment and an active spindle assembly checkpoint [142]. Consequently, long-term arrested cells will die in mitosis or slip through the spindle assembly checkpoint [141], leading to aneuploidy and cell death. Based on their target and mode of action, mitotic inhibitors, also referred to as cytoskeletal drugs, are grouped into four categories: (1) enhancers or stabilizers of actin polymerization (Jasplakinolide, Phalloidin); (2) preventing actin polymerization or sequestering actin dimers (Cytochalasin, Latrunculin, Swinholide); (3) enhancers or stabilizers of microtubule polymerization (Paclitaxel); 4) preventing or inducing microtubule polymerization (Colchicine, Nocodazole, Vinblastine, Rotenone, Demecolcine) [143]. Due to their clinical relevance, the microtubule-targeting drugs, Paclitaxel, Vinblastine, and Demecolcine, with broad indications, among others, in breast, ovarian, lung, pancreatic cancer or melanoma, will be in the focus for the remainder of this section.

Similar to other chemotherapeutic agents, known resistance mechanisms of mitotic inhibitors are diverse but include the removal of the drug by ABC transporter expression [128,144,145,146], changes in the drug target by differential isoform expression or mutation [147], alterations in microtubule-associated proteins (MAPs) that stabilize or destabilize microtubules (e.g., survivin, strathmin) [148,149], the activation of kinases (e.g., Aurora A or EGFRvIII) [150], the upregulation of anti-apoptotic components (e.g., Bcl-2 or Bcl-xL expression) and transcription factors (e.g., NF-κβ and STAT-3) [151,152,153,154,155], as well as an increased expression and the release of cytokines, such as IL-6 or IL-8 [156,157]. These resistance mechanisms are not exclusive and occur in combinations, e.g., acquired resistance to the microtubule-stabilizing therapeutic paclitaxel frequently coexists with a reduced susceptibility to inhibitors of EGFR–tyrosine kinases, leading to the selection of multidrug-resistant cancer cells [158].

With the advent of gene perturbation screening, resistance and susceptibility factors of mitotic drugs have been investigated at genome-scale. As such, Gerhards et al. performed an insertional mutagenesis screen in the haploid cell line, HAP1, to identify loci that, when interfered with, induce resistance to docetaxel. As expected, the group successfully identified ABCB1, the multidrug efflux transporter P-glycoprotein (P-gp), previously discussed to cause multidrug resistance [159]. Moreover, the same group identified CCNB1, MAD1L1, MAD2L1, and KNTC1, all of which contribute to the spindle-assembly-checkpoint, suggesting an abolished spindle-assembly-checkpoint to support mitotic slippage of cells arrested with docetaxel [159]. The White group used a genome-wide RNAi screening approach in combination with human NSCLC cells exposed to paclitaxel to identify synthetic lethal drug-gene combinations [160]. Confirming previous work, the group identified multiple proteins involved in microtubule function and dynamics, or associated with the gamma-tubulin ring complex [160], as well as a group of core proteasome components. Building on this work, the Neely group used HAP1 cells in combination with a CRISPR screening approach (GeCKO.v2 library) to identify resistance-causing loss-of-function mutations against paclitaxel, but extended their efforts to include an additional panel of 26 anticancer drugs. Among others, they identified the previously uncharacterized open reading frame C1orf115, containing a DUF4710 domain of unknown function, to convey robust resistance to paclitaxel, vincristine and the purine analog cladribine [161], suggesting C1orf115 to be an uncharacterized multidrug resistance gene that escaped previous identification. In contrast, Wei et al. used cervical squamous cell carcinoma cells and the GeCKO.v2 library to identify paclitaxel-sensitizing genes. As expected, they identified the ABC-transporter ABCC9 and an anti-inflammatory cytokine IL-37, plus several members of the PPP family of serine/threonine phosphatases (PPP1R7, PPP2R5B, PPP1R7, and PPP1R11) that, till then, were not associated with paclitaxel susceptibility [162]. Generating specific inhibitors against phosphatases is challenging, thus it will be interesting to identify downstream molecular targets of these phosphatases and elaborate their druggability to therapeutically harness this information. Genome-scale CRISPR screening in combination with transcriptomic analyses were used by the Shao group to explore paclitaxel resistance in triple-negative breast cancer cells. MITR, the truncated isoform of the histone deacetylase 9 (HDAC) was identified to cause paclitaxel resistance [163]. Mechanistically, the group could demonstrate that paclitaxel resistance is caused by an MITR-dependent counteraction of IL-11 transcription, with the ruxolitinib-mediated inhibition of JAK1/2 being able to reverse paclitaxel resistance in vitro and in vivo [163]. Alternative to gene inactivation by CRISPR knockout screens, Zhao et al. used a genome-wide CRISPR gene activation approach (SAM library) to identify gene-specific positive transcriptional changes mediating paclitaxel resistance in esophageal cancer. Interestingly, the group identified and validated the cyclin-dependent kinase inhibitor CDKN1A, the RNA-binding protein ELAVL2, and the cell surface glycoprotein TSPAN4 [164]. The group further supports their findings by demonstrating that the protein levels of CDKN1A, TSPAN4 and ELAVL2 can serve as risk factors for resistance to neoadjuvant chemotherapy in esophageal cancer [164], thus providing pre-clinical evidence for its therapeutic use.

In vitro conditions are currently the favored screening approach; however, in vivo conditions have also been applied to identify resistance and susceptibility factors for mitotic inhibitors. As such, the Patel group developed a prostate cancer orthograft model for in vivo CRISPR screening of paclitaxel susceptibility and identified the transcription elongation factor A (SII)-like protein, Tceal1, as a potential drug target [165]. Bulk RNA sequencing of Tceal1-depleted cells revealed the paclitaxel-susceptibility mechanism to be linked to the expression of genes required for the G2/M and G1/S phase cell cycle checkpoints [165]. While this is interesting and potentially identifies uncharacterized in vivo susceptibility factors, no previously described paclitaxel-susceptibility factors could be identified. It, therefore, remains to be tested if these differences account for the different model system used, or are potentially linked to technical challenges associated with genome-scale in vivo approaches. The above-discussed work primarily focused on identifying acute inhibitor resistance or susceptibility through unbiased in vitro gene perturbation approaches. However, characterizing chemotherapy resistance and its associated susceptibility mechanisms, developed through long-term drug exposure, may be of higher clinical relevance. These studies, however, remain sparse, as they generally require screening-amendable cell models with pre-existing drug resistances that take considerable time and efforts to generate. To close this gap, Gupta et al. generated paclitaxel-resistant breast cancer cell lines by continuous drug exposure and identified the resistance to be dependent on the expression of the EGF-receptor HER2 [166], a well-known drug target that is frequently amplified or overexpressed in breast cancer. In the future, it will, therefore, be crucial to expand unbiased genome-scale investigations to explore drug resistance and vulnerability mechanisms of mitotic inhibitors, selected for by continuous drug exposure.

### 3.4. Antimetabolites

Antimetabolite drugs are analogs of cell compounds that inhibit their formation or utilization. Ultimately, this leads to the inhibition of metabolic routes such as DNA synthesis, especially affecting fast-dividing cells. Antimetabolites can be classified into antifolates, ribonucleotide reductase (RNR) inhibitors, and nucleoside analogs. Antifolates (e.g., methotrexate) inhibit enzymes involved in folate metabolism such as DHFR, that are necessary for the synthesis of tetrahydrofolic acid and eventually lead to the impairment of thymidylic acid, purine synthesis and DNA replication [167]. The enzyme RNR, that is critical for the synthesis of nucleotide precursors for DNA synthesis, can be inhibited through two main mechanisms: the inhibition of the formation of an active form of RNR or inhibition of the enzyme once activated (reviewed in [168]). An example of the latter is hydroxyurea, a chemotherapeutic drug that also exhibits radiosensitizing effects. Nucleoside analogs act by inhibiting different enzymes involved in the generation of nucleotides or by mimicking physiological nucleosides so that their incorporation into DNA during replication results in chain termination and DNA synthesis impairment (reviewed in [169]). 

Gemcitabine (2′,2′-difluoro-2′-deoxycytidine; dFdC), a nucleoside analog of deoxycytidine that triggers cell cycle arrest and apoptosis, is the primary treatment option, alone or in combination with other drugs, in advanced pancreatic cancer, and is increasingly being used in other types of tumors [170,171,172,173,174]. Gemcitabine is a prodrug that requires metabolization to its active form gemcitabine triphosphate (dFdCTP). First, gemcitabine is transported into the cell mainly by the sodium-dependent membrane transporters hENT1 and hCNT1 (equilibrative and concentrative nucleoside transporters, respectively). Once in the cytoplasm, a series of phosphorylation events result in the formation of dFdCTP. The first phosphorylation event by dCK (deoxycytidine kinase) is considered to be the rate-limiting step. The most important mechanism of action of gemcitabine takes place through a process known as “masked chain-termination”. Once dFdCTP is incorporated into DNA by the DNA polymerase, only one additional nucleotide can be added, with the “hidden” dFdCTP remaining inaccessible to the DNA repair machinery. This precludes chain elongation and results in replication fork stalling, triggering cell cycle arrest and apoptosis. Gemcitabine also inhibits deoxynucleotide metabolism by reducing the dNTP pool by binding to RNR, which leads to a self-potentiation as gemcitabine becomes more likely to be incorporated into DNA (reviewed in [175]).

Despite its ability to inhibit tumor cell growth, intrinsic or acquired resistance to gemcitabine is frequently developed by patients after an initially good response. Several resistance mechanisms have been identified and include the deregulation of proteins involved in gemcitabine metabolism. The downregulation of the transporters hENT1 and hCNT1, or the enzyme dCK, as well as the overexpression of RRM1 and/or RRM2 (subunits of the RNR holoenzyme), have been observed in gemcitabine-resistant pancreatic tumor cells (reviewed in [176]). Interestingly, Nakano and colleagues found that a low ratio of hENT1, dCK/RRM1, and RRM2 gene expression, rather than the individual gene levels, was a characteristic of cells with acquired resistance to gemcitabine [177]. Low levels of Human antigen R (HuR), an RNA-binding protein that regulates dCK at the mRNA level, are also associated with gemcitabine resistance [178]. Furthermore, gemcitabine resistant tumor cells show alterations in different signaling pathways, including NF-κβ [179], AKT [180], MEK/MAPK [181], and HIF1α [182]. Epithelial-to-mesenchymal transition (EMT) [183] and miRNA delivery by exosomes also seem to contribute to gemcitabine resistance [184,185].

As discussed above, gene perturbation screens allow for the elucidation of molecular mechanisms responsible for resistance, so predictive biomarkers and/or drug-associated vulnerabilities can be exploited. In a systematic screen in the pancreatic cancer cell line, MiaPaCa2, in which 645 human kinases were inhibited by specific siRNAs, several kinases were found to increase gemcitabine-induced apoptosis when silenced [186]. The proteins with the most significant effect on cell survival upon gemcitabine treatment included members of the PI3K/AKT/mTOR signaling pathway, such as MAK, PAK, MAPKAP1, and PIK3CG, confirming the role of this survival pathway in gemcitabine resistance [187,188]. The checkpoint kinase 1 (CHEK1) was also among the significant hits. This is in agreement with its essential role in the signaling of the DNA damage and repair ATR-CHEK1 pathway. In line with this, Azorsa and colleagues also identified the silencing of CHEK1 in MiaPaCa2 cells to have a synergistic response with gemcitabine [189]. The involvement of the AKT-CHEK1 signaling pathway has been further confirmed by Fredebohm et al., who performed a synthetic lethal screen with an shRNA library targeting 10,000 genes in the pancreatic cell line BxPC-3, and also showed reduced cell viability in CHEK1-depleted cells treated with gemcitabine, suggesting that this mechanism may be present in a wider number of cell types. In the same study, the cell cycle checkpoint protein RAD17 was identified as the target with the highest effect, as its knockdown decreased cell viability of gemcitabine treated cells from an average of approximately 75% to 40% in the cell lines BxPC-3, MiaPaCa2, and JoPaca-1 [190]. RAD17 promotes cell cycle arrest through the AKT-CHEK1 pathway after the masked chain termination induced by gemcitabine. The knockdown of RAD17 in combination with gemcitabine treatment resulted in a forced cell cycle re-entry from S phase, ultimately leading to mitotic catastrophe and increased apoptosis. Furthermore, on the contrary to CHEK1, RAD17 inhibition does not have an antiproliferative effect on its own, suggesting that its pharmacological inhibition may have fewer undesirable effects on unperturbed cells.

Masoudi and colleagues performed a genome-wide CRISPR/Cas knockout screen using the GeCKO.v2 library in the pancreatic carcinoma cell line Panc1 [191]. This cell line has intrinsically higher gemcitabine resistance as compared to other pancreatic cells, such as the previously mentioned MiaPaCa2 and BxPC-3. Besides DNA repair, G2/M checkpoint and E2F targets, a Gene Set Enrichment Analysis (GSEA) revealed MYC targets to be important for gemcitabine treated Panc1 survival. The gene with the most prominent effect was SH3D21, as abolishing or inhibiting its expression resulted in an increased sensitivity to the drug. SH3D21 is a poorly studied gene, predicted to be involved in purine ribonucleoside triphosphate metabolism, but further studies would need to be conducted in order to confirm its role in gemcitabine resistance. Another interesting finding of this study was the enrichment of ERVFRD-1, a viral gene involved in endocytosis. To test whether ERVFRD-1 could be involved in gemcitabine uptake, the authors treated cells with an endocytosis inhibitor and found the effects of gemcitabine to be attenuated under this experimental condition, possibly suggesting another cellular uptake mechanism of gemcitabine. Intriguingly, this genome-scale screen did not identify other genes, such as the above-mentioned CHEK1, previously found in several studies, among the top candidates. This may be due to the different methodologies, with the CRISPR/Cas system being able to completely abolish gene function versus RNAi, where only the knockdown of a gene is achieved. The use of different cell models, with diverse degrees of inherent resistance to gemcitabine, might also explain the differences between studies.

As previously mentioned, epigenetic factors can also contribute to chemotherapy resistance. To investigate the role of genes involved in epigenetic control of chromatin regulation, Wei et al. conducted a CRISPR/Cas knockout screen and found the protein arginine methyltransferase gene 5 (PRMT5) to act as a synthetic lethal with gemcitabine in a patient-derived pancreatic cancer cell line [192]. PRMT5 is implicated in several functions, including genome organization, transcription, cell cycle, and spliceosome assembly. PRMT5 depletion results in excessive DNA damage accumulation, which leads to non-tolerable replication stress. Interestingly, PRMT5 expression is upregulated in pancreatic ductal adenocarcinoma (PDAC), and this enhanced expression is associated with poor patient survival. Moreover, PRMT5 is a druggable target, with ongoing clinical trials of small molecule inhibitors. The authors showed that the genetic depletion or pharmacological inhibition of PRMT5, together with gemcitabine treatment, results in a synergistic tumor growth inhibition.

Although gemcitabine has been traditionally used to treat pancreatic cancer, its employment in other types of malignancies has also increased. Indeed, the growing use of gemcitabine in other malignancies is also highlighted by studies using other cellular models. A loss-of-function screen in the cervical cancer cell line HeLa used an shRNA library to target kinases, phosphatases, tumor suppressor genes, DNA binding proteins, and modification enzymes. From the 13 candidate genes, only two were verified to cause resistance to gemcitabine treated cells. These were the splicing factors SRSF3 and SFPQ, involved in pre-mRNA splicing but also in other processes such as homologous DNA recombination and DNA damage response [193]. The authors did not follow up on investigating the mechanisms by which the downregulation of these proteins induced resistance to gemcitabine. However, the observation that the decreased expression of SFPQ and SRSF3 also induced resistance to Ara-C and 5-FU suggests that it might be through a shared molecular mechanism.

In a different study, a genome-wide CRISPR/Cas9 loss-of-function screen (Brunello library) was carried out in the gallbladder cancer (GBC) cell line NOZ, which displays high basal sensitivity to gemcitabine [194]. The authors found that the downregulation of the Elongator complex subunit 5 (ELP5) leads to gemcitabine resistance, and that ELP5 and U34 tRNA-modifying enzymes are required to induce cytotoxicity in GBC cells. This effect was further confirmed by in vivo experiments using xenograft models of ELP5-/- NOZ cells, where the gemcitabine-treated ELP5-/- group showed increased tumor growth compared with the WT group. Interestingly, treatment with gemcitabine seemed to induce an increased expression of different subunits of the Elongator complex. On the contrary, ELP5 depletion accelerated the degradation of some of those components, suggesting its role in maintaining the stability of the Elongator complex. The authors demonstrated that the loss of ELP5 impaired the expression of TP53 in a post-transcriptional manner, eventually leading to reduced levels of its downstream targets p21, BAX and cleaved caspase-3, and increased levels of anti-apoptotic Bcl-2, thus inhibiting apoptosis. Furthermore, exogenous ELP5 expression enhanced gemcitabine-induced apoptosis only in TP53 WT cells. Interestingly, the role of ELP5 as determinant or chemotherapy resistance might not be restricted to gemcitabine, as GSEA revealed that cisplatin and doxorubicin resistance signatures were also enriched in patients with low ELP5 expression. In addition, the dysregulation of TP53 and its downstream targets were also observed under cisplatin treatment. Therefore, although most unbiased gene perturbation screens were performed to identify gemcitabine resistance mechanisms in pancreatic cancer cell lines, it is of high clinical relevance to include additional cellular contexts to reveal common and context-specific genetic vulnerabilities.

## 4. Translating Chemoresistant Vulnerabilities—Conceptual and Technological Strategies

The majority of the studies presented in this review harness the concept of collateral sensitivity as a type of synthetic lethality that could be potentially exploited in cancer therapy. The idea that resistance towards a drug must inevitably come accompanied by an increased sensitivity to other compounds has led to the search of these acquired vulnerabilities in cancer cell lines resistant to chemotherapeutic agents, as well as to targeted drugs. Collateral sensitivities have been reported with cancer chemotherapeutics, including an increased sensitivity to microtubule inhibitors, cisplatin or cytarabine in paclitaxel-resistant CHO (Chinese hamster ovary) cells [195]. For example, Jensen et al. screened a panel of SCLC cell lines resistant to different chemotherapy agents and tested their sensitivity to 20 additional drugs [196]. With this approach, they identified cross-resistance as well as collateral sensitivities. Interestingly, vulnerabilities were found for all the resistant cell lines tested, one of them being the previously reported higher sensitivity to cisplatin in paclitaxel-resistant cells. MDR is often caused by the overexpression of drug efflux pumps, such as the ABC transporter MRP1, and this overexpression could itself be exploited as a collateral sensitivity that can be associated with the depletion of critical intracellular compounds such as glutathione, ATP and metals. MRP1-overexpressing cells usually show reduced levels of glutathione (GSH) [197,198,199], which can be additionally decreased using other therapeutic agents, such as verapamil [200,201], that further increase GSH efflux, leading to oxidative stress and apoptosis [202,203]. Oxidative stress induced by high levels of ROS is characteristic of chemotherapy-treated cells [187,204,205], and several redox-regulating enzymes could be potentially targeted, including mitochondrial electron transporters and GSH metabolism-related enzymes (reviewed in [206]). Another approach to exploit this acquired vulnerability is the “one-two-punch” strategy, elaborated by the Bernards’ group [5]. Similar to chemotherapy-resistant cells, melanoma cells resistant to the BRAF inhibitor vemurafenib have increased levels of ROS, which can be additionally increased by using the histone deacetylase inhibitor vorinostat. This sequential treatment leads to much higher levels of ROS in the resistant cells, causing severe DNA damage and apoptosis. The clinical efficacy of the “one-two-punch” hypothesis is currently evaluated with vorinostat treatment in patients with resistant BRAF V600E mutated advanced melanoma [207], and the results will hopefully prove that the concept is clinically relevant.

These discussed studies also demonstrate the usefulness of genetic perturbation screens in the investigation of acquired vulnerabilities in chemotherapy-resistant cancer cells. Many of the hits found to act synthetic lethal with the drugs in the resistant cells pertain to cell proliferation, DNA damage repair or the apoptosis pathways, which makes them potentially targetable with inhibitors of these signaling pathways. For instance, CHEK1 is a commonly found hit in screens that search for genes that could render cells more susceptible to gemcitabine treatment when downregulated. In fact, it has been shown that CHEK1 can be targeted with the inhibitor AZD7762 to sensitize pancreatic cancer cell lines to gemcitabine both in vitro and in vivo [208,209]. Apoptotic pathway alteration is another commonly found feature in chemoresistant cells, usually evidenced by an increased expression of the anti-apoptotic protein Bcl-2 [194,210,211]. Under chemotherapy selective pressure, tumor cells can become “addicted” to Bcl-2 expression for survival, so an obvious strategy to overcome this resistance mechanism may be the use of Bcl-2 inhibitors (reviewed in [212,213]). Pearce et al. showed that the increased expression of Bcl-2 in paclitaxel-resistant lung cancer cells could be exploited by a different approach [214]. Bcl-2 can be converted from an anti-apoptotic protein to a pro-apoptotic one through interaction with Nur77 in the mitochondria. The authors demonstrated that Bcl-2 can be pharmacologically targeted with a peptide that mimics Nur77, preferentially inducing apoptosis in Bcl-2 overexpressing paclitaxel-resistant cells and inhibiting tumor growth in a zebrafish xenograft model.

Most unbiased gene perturbation efforts investigate what would resemble intrinsic resistance mechanisms in the sense that the genetic perturbation is introduced prior to short-term exposure to a given drug. However, searching for targetable collateral sensitivities that emerge as a result of long-term drug exposure might be more beneficial, as these model system likely more accurately mimic the heterogenous chemoresistance profiles found in patients. It is also of great importance to be able to prioritize cancer therapeutic targets, and this should be done on the basis of context-penetrating mechanisms of resistance and/or vulnerabilities. In an effort to link drug response to genomic alterations, Garnett et al. performed a systematic screen in a panel of 639 cancer cell lines with 130 targeted agents and chemotherapeutic drugs [215]. As expected, strong associations were found for oncogenes that are targets of their specific drug, but they also found marked sensitizing genotypes in small subsets of cell lines, highlighting the need for large panels of cell lines to uncover potentially relevant genomic features. To address the above-mentioned aspects, a larger number of drug-resistant cell lines pertaining to different cancer entities should be investigated systematically, with the “The Resistant Cancer Cell Line (RCCL) collection” representing a valuable resource that awaits its comprehensive genetic characterization (https://www.wass-michaelislab.org/rccl.php). Another important aspect is that resistance rarely emerges from a single mechanism. More often, several co-occurring gain-of-function and/or loss-of-function mechanisms synergistically contribute to a patient’s chemoresistance profile. In this context, technologies for the robust performance of genetic multiplexing approaches for the simultaneous perturbation of multiple loci are needed [73,216]. While these combinatorial approaches gain momentum in the identification of synthetic gene interactions, their general application and analysis are far from standardized. However, these approaches hold great potential to uncover druggable vulnerabilities otherwise missed in monogenic screens due to functional buffering effects [217].

## Figures and Tables

**Table 1:** Strengths and weaknesses of RNAi and CRISPR technologies.

	RNAi	CRISPR
Strengths	Selective targeting of different transcripts of the same gene [67] Independent of cell ploidy or chromatin conformation [68] Knockdown effect is more similar to drug effect [68] Can target any mRNA sequence [68]	High knockdown efficiency [69] Identification of essential genes with low expression [55,70] High on-target rates [48,50] Targeting of non-transcribed elements [60,63] Low immune response [71,72] Stable gene disruption [51] Highly modular system (dCas9 with effector domains) [34] Pooled combinatorial designs available [73]
Weaknesses	Considerable off-target effects [48,49] Incomplete silencing of the target gene (knockdown) [52] Stimulate immune response [53,54] Transient gene silencing [74]	Residual protein expression (incomplete KO/truncated transcripts) [75,76] Target space depends on PAM [57] Stochastic mutational outcome (dependent on DNA repair) [77] Depends on chromatin structure and ploidy of the cell [47] Requires expression of a Cas-enzyme [20,29] Induces DNA damage (DSB) [58,59] Performance is influenced by a cells’ TP53 status [78,79,80]

## Data Availability

All data are contained within the article.

## Supplementary Materials

The following are available online at https://www.mdpi.com/2073-4409/10/2/260/s1, Table S1: Chemoresistant gene perturbation screens.
